# Hepatitis B virus infection among people who use drugs in Iran: a systematic review, meta-analysis, and trend analysis

**DOI:** 10.1186/s12954-020-00424-w

**Published:** 2020-10-21

**Authors:** Yasna Rostam-Abadi, Hossein Rafiemanesh, Jaleh Gholami, Behrang Shadloo, Masoumeh Amin-Esmaeili, Afarin Rahimi-Movaghar

**Affiliations:** 1 Iranian National Center for Addiction Studies (INCAS), Tehran University of Medical Sciences, No. 486, South Karegar Ave., 1336616357 Tehran, Iran; 2 Department of Epidemiology, School of Public Health and Safety, Shahid Beheshti University of Medical Sciences, Tehran, Iran; 3 Mental Health Department, Johns Hopkins Bloomberg School of Public Health, Baltimore, USA

**Keywords:** Hepatitis B, Blood-borne infections, Substance use, Addiction, Harm reduction, Injecting drug use, Epidemiology, Iran

## Abstract

**Background:**

People who use drugs (PWUD) are considered as one of the main at-risk populations for Hepatitis B virus (HBV) infection. We conducted a systematic review on the prevalence of HBV infection among PWUD in Iran.

**Methods:**

Consistent with PRISMA guideline, international (Medline, Web of Science, Scopus, and Embase) and national (Scientific Information Database) databases were searched using a comprehensive search strategy up to September 2019. The retrieved records were reviewed, and experts were contacted for unpublished studies. Studies on Iranian PWUD reporting HBV surface Antigen (HBsAg) prevalence among people who inject drugs (PWID) and non-injecting PWUD were included. HBsAg prevalence was pooled for PWID and non-injecting PWUD and for other subgroups using random-effects model meta-analysis. The trend of HBV prevalence over time was investigated using meta-regression analysis.

**Results:**

Overall, 35 studies reported data on HBV infection among PWID (33 studies) and non-injecting PWUD (11 studies). The pooled prevalence of HBsAg among PWID was 4.8% (95% CI 3.7–6.2). The only risk factor significantly associated with the odds of positive HBsAg in PWID was the previous history of imprisonment (OR 1.72, 95% CI 1.29–2.30, *p* value = 0.000). The pooled estimate of HBsAg among non-injecting PWUD was 2.9% (95% CI 2.5–3.2). Time trend analyses showed significant decrease in HBV prevalence among PWID reaching from 8.2% (95% CI 3.9–16.5) in 2004–2006 to 3.1% (95% CI 2.3–4.1) in 2016 and later (b = -0.07; *p* value = 0.05). No significant trend was detected for non-injecting PWUD.

**Conclusion:**

The prevalence of HBV infection among non-injecting PWUD and even PWID was not considerably higher than the Iranian general population. This might be the result of extensive harm reduction interventions in Iran. However, it seems that there are subgroups of PWID, who do not adequately benefit from existing harm reduction interventions. Future programs should more specifically target these high-risk groups.

## Introduction

Hepatitis B virus (HBV), a vaccine-preventable infection, remains one of the leading causes of acute and chronic liver diseases [1]. In 2017, there were 13.8 million new cases of chronic HBV globally [2]. The resulting cirrhosis and hepatocellular carcinoma were the main contributing factors to HBV burden. In 2017, HBV-related DALY and mortality rate were estimated as 107.2 years and 3.7 per 100,000 in Iran, respectively [2].

Hepatitis B is one of the main infectious diseases among people who use drugs (PWUD). There are several reasons why PWUD are considered more vulnerable to HBV. Needle sharing is one of the major routes of transmission among people who inject drugs (PWID) [3]. Substance use contributes to other certain vulnerabilities [4] such as homelessness [5, 6], incarceration [7], and unsafe sexual contacts [1]. In addition, this population has limited access to health services required for timely prevention, diagnosis, and treatment [3]. The World Health Organization (WHO) considers PWUD as one of the main adult target populations in its strategy for ending HBV [1]. Promoting harm reduction services is introduced as one of the five core interventions for combating viral hepatitis, including HBV; alongside with vaccination, prevention of mother-to-child transmission, safety of medical interventions, and treatments programs [1]. The impact targets for 2030 by the WHO are 90% of reduction in new chronic cases of HBV and 65% reduction in attributed mortality [1].

Substance use has been a public health issue for decades in Iran [8]. The Iranian National Mental Health Survey (IranMHS) estimated the prevalence of any substance use disorder to be 2.8% in the Iranian general population [9]. Although it was previously estimated that more than 20% of PWUD had used drugs through injection in the last 12 months [10], recent studies have shown that the figure has decreased to about 3% [11, 12]. In Iran, drug use and injecting drug use are known as the main risk factors for HIV and HCV [13–17]; therefore, the country has adopted extensive harm reduction measures to control blood-borne infections among PWID for more than a decade.

HBV prevalence among PWUD has been assessed in many studies in Iranian population, and the results were quite different. In addition, HBV prevalence among PWID was assessed in a global systematic review. The provided estimate for HBV infection among Iranian PWID was considerably lower than the corresponding global (9%) and regional Figures (8.1%). However, this was not the case for HIV and HCV estimates [4]. Therefore, in order to provide a more detailed picture of HBV among PWUD in Iran, we conducted a systematic review on all studies providing the prevalence of HBV surface antigen (HBsAg) among both PWID and non-injecting PWUD, according to the socio-demographic characteristics, recruitment settings, high-risk behaviours, and geographical distribution. HBsAg, a surface protein, can be detected in both acute and chronic HBV infection, also indicating that the individual is infectious [18]. We also investigated the trend of changes in HBV prevalence among PWID and non-injecting PWUD over time.

## Methods

### Search strategy and selection criteria

The method used in this study is in accordance with the PRISMA guideline. For finding published studies, we searched international (Medline, Web of Science, Scopus, and Embase) and Iranian (Scientific Information Database—SID) bibliometric databases using a comprehensive search strategy in September 2019. Search strategy terms were categorized in four groups and combined using Boolean operators: (1) keywords related to Iran, including names of cities, provinces, and major universities in Iran; (2) the names of substances used in Iran and terms related to drug use or drug use disorders; (3) terms related to hepatitis and HIV. No restrictions on publication date and language of full text were applied. We completed our search by reviewing the references of the retrieved studies (backward citation tracking) and contacting experts in this field in order to access unpublished studies.

Studies were included if they: (1) were cross-sectional or cohort studies; (2) were on human subjects; (3) had been done on Iranian population; (4) were conducted on a target population of those with drug use or drug use disorders; (5) had assessed HBsAg prevalence (acute or chronic infection); (6) provided HBV or any index of HBV prevalence by injecting and non-injecting drug use group; and (7) had not recruited the study samples from infectious disease wards or HIV treatment and care centres. Due to the higher prevalence of infectious diseases in those admitted to infectious disease wards, the recruited sample would not be generalizable to PWUD and would be biased for estimating prevalence of HBV infection.

### Screening, data extraction, and quality assessment

Screening of the identified documents was conducted in two stages: (1) screening of titles and abstracts to exclude irrelevant studies, and (2) assessing full texts for eligibility and inclusion criteria. If HIV or HCV prevalence and not HBV were stated in the abstract, the study was not excluded and full-text was reviewed as well. Both stages were done independently by two reviewers, and discrepancies between the reviewers were resolved by the third reviewer. The third reviewer also randomly checked both included and excluded records at each stage.

Two investigators independently extracted the data of included studies using a data extraction sheet. The extracted data of each study were checked by the two reviewers and discussed in case of disagreements. Data extraction sheet included bibliometric characteristics of the citation, year of study implementation, recruitment setting (prison, drug treatment centres, drop-in centres (DICs), etc.), study location (province), type of biological test for HBV, co-infection with HIV and HCV, route of drug use (injecting and non-injecting), definition of injecting drug use, sampling method, sample size, response rate, socio-demographic characteristics of the participants, history of high-risk behaviours, test results, and gender-specific data.

The quality of each study was assessed by an 8-item critical appraisal form, which was adapted from Joanna Briggs Institute (JBI) critical appraisal-checklist for cross-sectional studies [19] in our research centre and has been used in similar studies [20]. If the data on four of these items (source of the sampling, subgroup analysis for gender, the type of laboratory test, and the year of the study) were not provided in the document, the authors were contacted for obtaining the related data. However, we reported that item as unfulfilled criteria in any case.

### Statistical analyses

After entering the extracted data into an excel sheet, the R version 3.5.3 was used for statistical analyses. For estimating the pooled prevalence, we used the "metaprop" command and random-effects model for estimating the pooled prevalence of HBsAg, separately in PWID and non-injecting PWUD, and also in different subgroups of drug users according to socio-demographics, recruitment settings, and high-risk behaviours. The "metabin" command was used to calculate the DerSimonian-Laird pooled odds ratios for detecting association between various risk factors and HBsAg prevalence. The prevalence of HBsAg by province was presented in a map using the Arc GIS software version 10.5.

The number of studies conducted in each year was few; therefore, the prevalence of HBsAg for every three years was pooled and depicted in a line graph for showing the trend of prevalence over the years, and because of the nonlinear trend, we fitted meta-regression line for the segments to evaluate the significance of the line’s slope. I-squared and Tau-squared statistics were applied for heterogeneity assessment. For heterogeneity interpretation, we used the following thresholds for I2 being 50–90% as may represent substantial heterogeneity; and 75–100% as considerable heterogeneity [21]. Meta-regression, using the "metareg" command, was performed perusing the source of heterogeneity among the included studies.

## Results

### Study selection

**Fig. 1:**
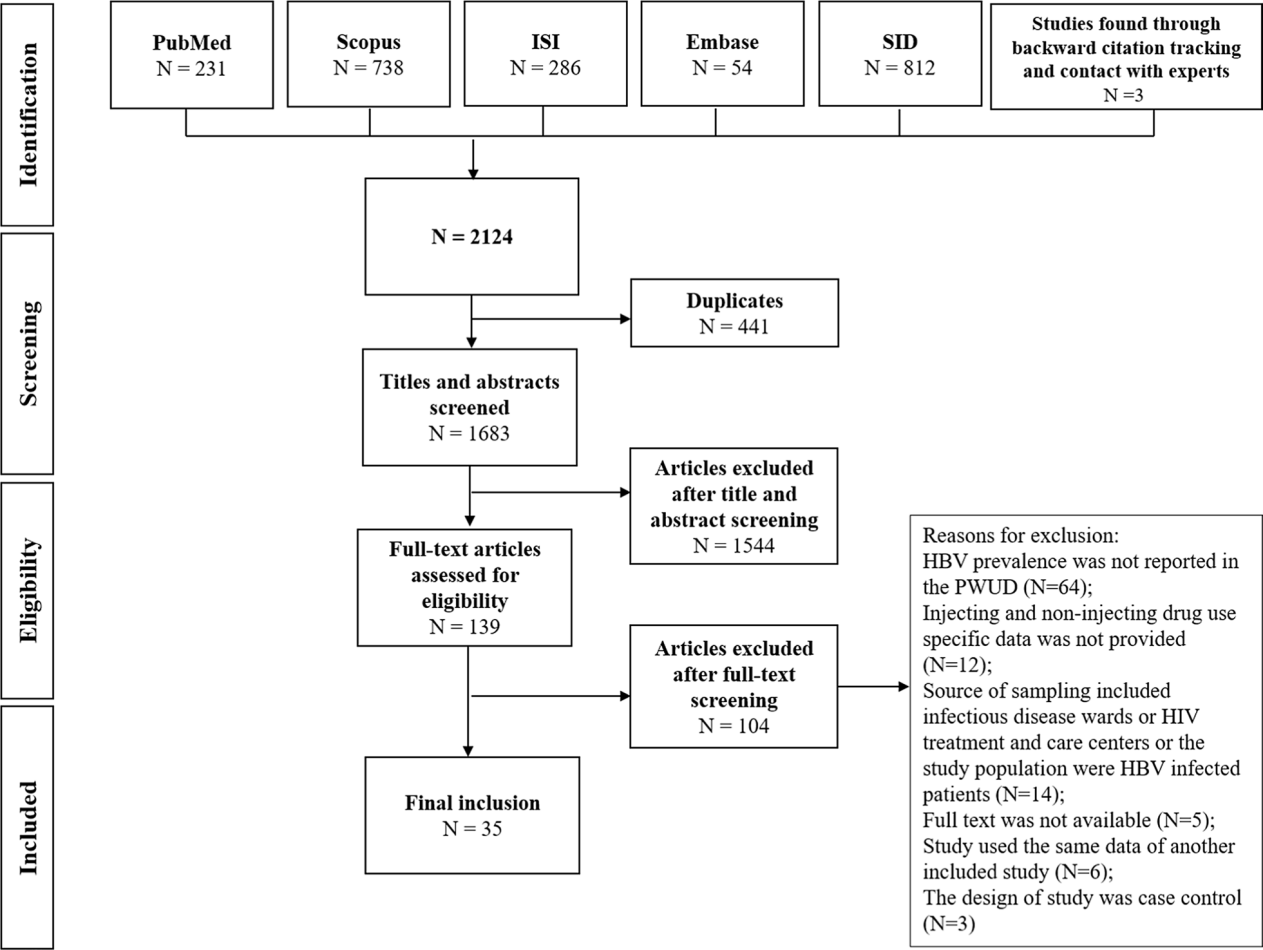
Flow diagram of the study selection

From a total of 2124 citations, 2121 records were found through electronic search in the online databases and three studies were identified through backward citation tracking and contacts with experts. After removing the duplicates, the title and abstract of the 1683 studies were screened. There were 136 records eligible for full-text review. Finally, 35 studies were included in the review (Fig. 1).

### Study characteristics

A total of 35 studies conducted on 18,631 PWUD from the year 1990 to 2017 were included in this systematic review. Of those, 33 studies included PWID (*N* = 9805) and 11 studies included non-injecting PWUD (*N* = 8826). There were nine studies providing estimates for both PWID and non-injecting PWUD. The total sample size ranged from 29 to 4614 among different studies. Four studies were multi-provincial, and other studies were implemented in 13 different provinces. Based on the recruitment setting, studies fell into 6 different categories: prisons (15 studies, *N* = 12,900), treatment centres (7 studies, *N* = 880), communities (4 studies, *N* = 1882), DICs (4 studies, *N* = 1003), mixed settings (4 studies; *N* = 1840), and hospital (one study; *N* = 126). Eight studies defined injecting drug use as any lifetime injection. On the other hand, there were studies which defined injecting drug use as usual route of drug use (*N* = 1), current injecting drug use (*N* = 1), history of injection in the last month (*N* = 1), history of injection in the last two months (*N* = 1), history of injection in the last three months (*N* = 2), history of injection in the last 12 months (*N* = 2), or the presence of injection marks (*N* = 2). All these latter 10 studies were merged into a category of "current PWID" in the analyses. Fifteen (45.5%) studies had not provided a definition for injecting drug use. Seven studies included both male and female PWUD, 13 studies only males, 2 studies only females, and in the other 13 studies, the gender of participants was not reported. The mean age of the subjects ranged from 28.8 to 48.2 years among the 16 studies providing this measure.

Any history of high-risk injection, such as ever sharing a needle, was present in 11.0% to 76.6% of the PWID among 15 different studies. Eight studies reported high-risk sexual behaviours among PWID with different definitions (men having sex with men, having a PWID partner, extramarital relationship, extramarital relationship without protection, sex exchange for money or drug, having sex with a sex worker, and multiple partners). High-risk sexual behaviours ranged from 5.0% to 43.1%; no study assessed related measures among non-injecting PWUD. Eight studies reported a history of tattooing, ranging from 27.7% to 78.0% among PWID.

Excluding 14 studies recruiting their sample from prison, history of previous imprisonment ranged from 35.3% to 77.6% among PWID in nine different studies. History of previous imprisonment among non-injecting PWUD was reported in a single study and was 1.3%. Further details on the studies' and participants' characteristics are depicted in Tables 1, 2 and 3.

### Quality assessment

Critical appraisal of the included studies using an 8-item tool showed that the number of unfulfilled criteria among studies ranged from zero in three studies to 4 or more (low-quality studies) in nine studies (Table 1). The mean (SD) and median of the unfulfilled criteria among studies were 2.5 (1.4) and 3.0, respectively. The most prevalent items were not providing the response rate (*N* = 25), method of sampling not being random or census (*N* = 23), and not providing gender-specific data (*N* = 17).

**Table 1:** Characteristics of the included studies on HBV prevalence among people who use drugs in Iran

No	Author, year	Year of study implementation	Province(s)	Recruitment setting	Sampling method	Sample size (male, female)	Target population	Definition of PWID	Response rate (%)	Unfulfilled quality criteria
1	Asadollahi, 2019 [47]	2017	Khuzestan	One DTC	UK	131 (UK)	PWID	UK	UK	2, 3, 4, 5, 6
2	Gheibipour, 2019 [48]	2016	Kermanshah	Two DICs and high-risk areas	Snowball sampling	606 (606,0)	PWID	UK	UK	2, 3, 5
3	Moradi, 2019 [49]	2016	National (10 provinces)	29 prisons	Random multistage sampling	4614 (UK)	PWID, non-injecting PWUD	Lifetime	98.1	4
4	Moradi, 2018 [50]	2015	National (9 provinces)	26 prisons	Random multistage sampling	4078 (UK)	PWID, non-injecting PWUD	Lifetime	88.8	4
5	Ziaee, 2016 [51]	2013–2014	South Khorasan	Household	UK	148 (UK)	PWID; non-injecting PWUD	Lifetime	UK	2, 3, 4, 5
6	Kandelouei, 2013 [52]	2013	Tehran	Three DICs in different areas with high rates of high-risk behaviours	UK	129 (128, 1)	PWID	Current	UK	2, 3
7	Moezzi, 2014 [53]	2013	Chaharmahal and Bakhtiari	Household	Cluster sampling	29 (UK)	PWID	UK	UK	3, 4, 5
8	Nokhodian, 2014 [54]	2012	Isfahan	Two prisons	Census	970 (970,0)	PWID	Lifetime	UK	2, 3
9	Ramezani, 2014 [55]	2012	Markazi	An MMT centre	UK	100 (100,0)	PWID	Last 3 months	UK	2, 3
10	Mohammadkhani-Ghiasvand, 2016 [56)	2010–2011	Tehran	An MMT centre	Census	220 (UK)	PWID; non-injecting PWUD	UK	UK	3, 4, 5, 6
11	Alipour, 2013 [57]	2010	Tehran, Khorasan Razavi, Fars	Four DICs	Convenient	42 (0, 42)	PWID	Lifetime	UK	2, 3
12	Alipour, 2013 [57]	2010	Tehran, Khorasan Razavi, Fars	Four DICs	Convenient	226 (226,0)	PWID	Last 12 months	UK	2, 3
13	Momen-Heravi, 2013 [58]	2010	Isfahan	Several MMT clinics, one DIC, an HCT centre	Convenient	300 (288, 12)	PWID	UK	UK	2, 3, 5
14	Teimori, 2011 [59]	2010	Kermanshah	One DTC	Census	76 (0, 76)	PWID; non-injecting PWUD	Usual route	UK	3
15	Khosravani, 2012 [60]	2009–2010	Kohkiloyeh & Boyerahmad	Two DTCs, one prison, 4 wards from two hospitals	UK	158 (157, 1)	PWID	Lifetime	UK	1, 2, 3, 4
16	Ziaee, 2014 [61]	2009–2010	South Khorasan	Three prisons	Stratified random	59 (UK)	PWID	UK	UK	2, 3, 4, 5
17	Khodadoostan, 2014 [62]	2009	Isfahan	NR	Announcement-based	1588 (UK)	PWID	Lifetime	NA	2, 4, 7
18	Nokhodian, 2012 [63]	2009	Isfahan	Central prison	Census	49 (0, 49)	PWID, non-injecting PWUD	Lifetime	100	None
19	Sofian, 2012 [64]	2009	Markazi	Prisons and mandatory residential centre for drug addiction	Census	153 (153,0)	PWID	Last 3 months	100	None
20	Khajedaluee, 2016 [65]	2008	Khorasan Razavi	Two prisons	Stratified random	606 (UK)	PWID, non-injecting PWUD	UK	UK	3, 4, 5
21	Zamani, 2010 [66]	2008	Isfahan	One DIC, at parks and streets	Respondent driven	117 (114, 3)	PWID	Last month	99.2	2, 4
22	Radfar, 2008 [67]	2007	Isfahan	A residential short-term rehabilitation centre	Convenient	40 (40,0)	PWID	UK	UK	2, 3, 4, 5, 6
23	Rahimi-Movaghar, 2010 [68]	2006–2007	Tehran	Three DTCs, 2 DICs, public places in 5 high-risk areas	Consecutive and purposive sampling using ethnographic observations, peer referral, snowball sampling	864 (827, 37)	PWID	Last 2 months	99.4 (for blood samples)	2, 3
24	SeyedAlinaghi, 2010 [69]	2006	Tehran	A mandatory rehabilitation centre	Census	452 (452,0)	PWID	Urine test and injection marks	90.6	None
25	Mardani, 2009 [70)	2004–2005	Ghom	One prison	UK	808 (UK)	PWID; non-injecting PWUD	UK	UK	2, 3, 4, 5
26	Talaei, 2007 [71]	2004–2005	Tehran	One poisoning hospital	Census	126 (UK)	non-injecting PWUD	Lifetime	UK	3, 4
27	Taghizadeh Asl, 2013 [72]	2003–2005	Alborz	Triangular clinic in a prison	Convenient	132 (132,0)	PWID	UK	88	2, 5, 6
28	Azarkar, 2007 [73)	2004	South Khorasan	One prison	Stratified random	140 (UK)	non-injecting PWUD	UK	UK	3, 4, 5
29	Imani, 2008 [74]	2004	Chaharmahal & Bakhtiari	One DTC	Convenient	133 (131, 2)	PWID	UK	UK	2, 3, 5
30	Khodadadizadeh, 2006 [75]	2003	Kerman	One DTC	Convenient	180 (172, 8)	PWID; non-injecting PWUD	UK	UK	2, 3, 4, 5
31	Pourahmad, 2007 [76]	2003	Isfahan, Chaharmahal & Bakhtiari, Lorestan	Four prisons	UK	401 (401,0)	PWID	UK	UK	2, 3, 5
32	Davoodian, 2009 [77]	2002	Hormozgan	Two prisons	Random	252 (252,0)	PWID	UK	UK	3, 4, 5
33	Tavakkoli, 2008 [78]	2001–2002	Tehran	Two prisons and 3 DTCs	Prisons: Random DTCs: Consecutive	518 (464, 54)	PWID	Regular injection for at least one year	98.5	2
34	Rowhani-Rahbar, 2004 [79]	2001	Khorasan Razavi	One prison	Convenient	101 (101,0)	PWID	Injection scars and identified as PWID by health personnel	92.7	2
35	Masaud, 1996 [80]	1990–1991	Tehran	Two prisons	UK	88 (48, 40)	PWID	Lifetime	UK	1, 2, 3, 7

Numerals of unfulfilled criteria; (1) Source of sampling was not well-presented or not representative of the target population; (2) The method of sampling was not appropriate; (3) The response rate was not provided or was under 70% or the non-responders were different from respondents in the main demographic characteristics; (4) Subgroup analyses were not performed for gender; (5) The study subjects (definition of injecting and non-injecting drug use) and the setting were not described in detail; (6) The condition was not measured by a valid method; (7) The year of the study was not stated; (8) The sample size was not adequate

*UK* unknown, *PWID* people who inject drugs; Non-injecting *PWUD* non-injecting people who use drugs, *DTC* drug treatment centre, *DIC* drop-in centre

### HBV prevalence among PWID and subgroups.

**Fig. 2:**
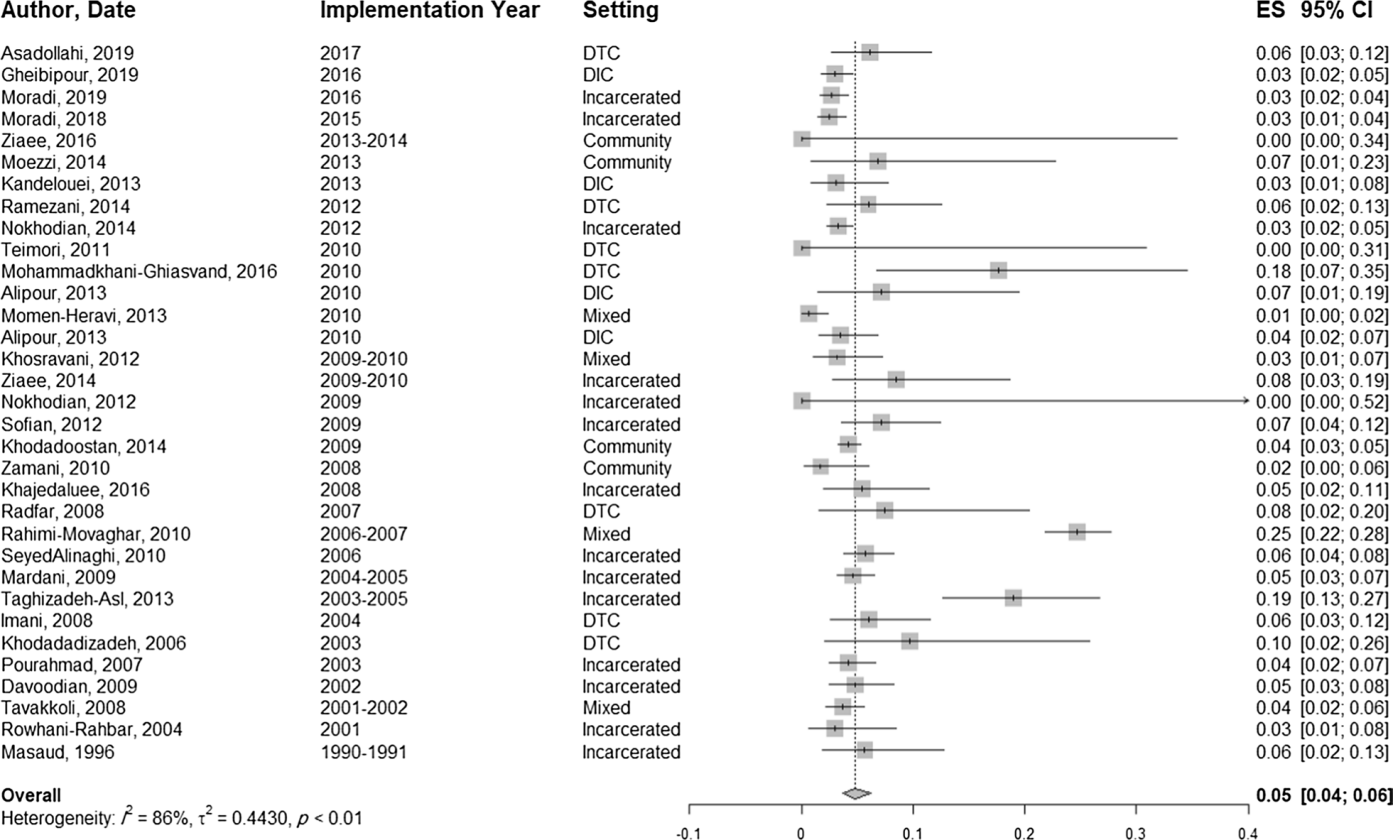
The pooled prevalence of HBV among PWID

The prevalence of HBsAg among PWID ranged from 0% to 24.6% in different studies (Table 2). The pooled prevalence of HBsAg positive cases among PWID was estimated to be 4.8% (95% CI 3.7–6.2; *I*^2^ = 86%, 33 studies, *N* = 9805) (Fig. 2). The pooled prevalence was 5.1% (95% CI 3.3–7.7, *I*^2^ = 92.9%, 20 studies, *N* = 5621) in male PWID and 2.9% (95% CI 0.4–17.2, *I*^2^ = 77.5%, 9 studies, *N* = 199) in female PWID.

**Table 2:** Findings of studies on HBV prevalence among people who inject drugs in Iran

No	Author, Year	Recruitment setting	Age characteristics	History of ever sharing needle equipment (%)	History of other high-risk injection (%)	History of incarceration (%)	History of a high-risk sexual relationship (%)	Tattooing/ Cupping (%)	Sample size (Male, Female)	HBsAg positive cases (%)			Other tests (%)
										Total	Male	Female	
1	Asadollahi, 2019 [47]	DTC	Mean (SD): 48.2 (10.4) Range: 29–71	–	–	–	–	–	131 (UK)	8^a^ (6.1)	–	–	–
2	Gheibipour, 2019 [48]	DIC	Mean (SD): 36.7 (8.5) Range:18–65	–	–	77.6	–	Tattoo: 62.1	606 (606,0)	18 (2.9)	18 (2.9)	–	–
3	Moradi, 2019 [49]	Incarcerated	–	36.0	–	NA	–	–	697 (UK)	19 (2.7)	–	–	–
4	Moradi, 2018 [50]	Incarcerated	–	29.3	–	NA	–	–	678 (UK)	17 (2.5)	–	–	–
5	Ziaee, 2016 [51]	Community	–	–	–	–	–	–	9 (UK)	0 (0)	–	–	–
6	Kandelouei, 2013 [52]	DIC	30–40 years: 34.6%	25.0	–	68.3	–	Tattoo:38.8	129 (128, 1)	4 (3.1)	4 (3.1)	0 (0)	–
7	Moezzi, 2014 [53)	Community	> 15	–	–	–	–	–	29 (UK)	2 (6.8)	–	–	–
8	Nokhodian, 2014 [54]	Incarcerated	Mean (SD): 32.6 (8.1)	–	Injection in prison: 40.3	NA	MSM:43.1	–	970 (970,0)	32 (3.3)	32 (3.3)	–	HBcAb: 127 (13.0); Isolated HBsAg: 120 (12.3)
9	Ramezani, 2014 [55]	DTC	Range: 17–58	54.0	Unsafe injection in prison:25.0; First injection i* N* < 18: 24.0	73.0	MSM: 9.0; With PWID: 14.0	Tattoo: 78.0	100 (100,0)	6 (6)	6 (6)	–	HCV: 6 (6); HCV-HIV: 5 (5)
10	Mohammadkhani-Ghiasvand, 2016 [56]	DTC	–	–	–	–	–	–	34 (UK)	6 (17.6)	–	–	–
11	Alipour, 2013 [57]	DIC	Mean (SD): 33.0 (1.0)	55.0	–	–	–	–	42 (0, 42)	3 (7.3)	–	3 (7.3)	–
12	Alipour, 2013 [57]	DIC	Mean (SD): 37.0 (1.1)	39.1	–	–	–	–	226(226,0)	8 (3.6)	8 (3.6)	–	–
13	Momen-Heravi, 2013 [58]	Mixed	Mean (SD): 34.9 (9.7)	11.0	–	76.7	MSM: 20.7; Extramarital: 20.3	Tattoo: 41.0; Cupping: 24.7	300 (288, 12)	2 (0.7)	2 (0.66)	0 (0)	–
14	Teimori, 2011 [59]	DTC	Mean: 35.2 Range: 20–54	–	–	–	–	–	10 (0,10)	0 (0)	–	0 (0)	–
15	Khosravani, 2012 [60]	Mixed	–	–	–	–	–	–	158 (157, 1)	5 (3.2)^b^	5 (3.2)^b^	0 (0)^b^	–
16	Ziaee, 2014 [61]	Incarcerated	–	–	–	NA	–	–	59 (UK)	5 (8.9)	–	–	–
17	Khodadoostan, 2014 [62]	Community	–	–	–	–	–	–	1588 (UK)	67 (4.2)	–	–	–
18	Nokhodian, 2012 [63]	Incarcerated	–	–	–	NA	–	–	5 (0, 5)	0 (0)	–	0 (0)	HBcAb: 2 (40); HBsAb: 2 (40)
19	Sofian, 2012 [64]	Incarcerated	Mean (SD): 30.7 (5.9)	–	–	NA	–	–	153 (153,0)	11 (7.2)	11 (7.2)	–	HCV: 9(5.9) HIV: 13(2.0) HCV-HIV: 2(1.3)
20	Khajedaluee, 2016 [65]	Incarcerated	–	–	–	NA	–	–	111 (UK)	6 (5.4)	–	–	–
21	Zamani, 2010 [66]	Community	Mean (SD): 29.0 (6.6)	31.2	–	71.2	MSM: 11.3 Exchanged for money or drug: 24.9	Tattoo:55.8	117 (114, 3)	2; Estimated RDS measure: 0.7	–	–	–
22	Radfar, 2008 [67]	DTC	Mean (SD): 28.8 (6.2)	–	–	–	–	–	40 (40,0)	3 (7.5)	3 (7.5)		–
23	Rahimi-Movaghar, 2010 [68]	Mixed	Mean (SD): 33.9 (9.4)	76.6	Unsafe injection in prison: 15.2; Any sharing in the L6M: 63.9	70.9	Extramarital without protection in the L6M: 36.4	–	864 (827, 37)	213 (24.7)	385 (46.5)^b^	13 (35.1)^b^	HCV: 181(21.0) HIV: 67(7.8) HCV-HIV: 56(6.5)
24	SeyedAlinaghi, 2010 [69]	Incarcerated	25–34 years: 51.7%	27.2	Unsafe injection in prison: 6.2	74.3	MSM: 5.0; With sex worker: 23.3	Tattoo: 27.7	452 (452,0)	26 (5.8)	26 (5.8)	–	–
25	Mardani, 2009 [70]	Incarcerated	–	–	–	NA	–	–	644 (UK)	30 (4.7)	–	–	–
26	Taghizadeh Asl, 2013 [72]	Incarcerated	Mean (SD): 31.4 (8.2)	–	–	NA	–	–	132 (132,0)	25 (18.9)	25 (18.9)		–
27	Imani, 2008 [74]	DTC	Mean (SD): 31.3 (7.1)	19.6	–	35.3	–	–	133 (131, 2)	8 (6.0)	8 (6.1)	0 (0)	–
28	Khodadadizadeh, 2006 [75]	DTC	–	–	–	–	–	–	31 (31, 0)	3 (9.7)	3 (9.7)	–	–
29	Pourahmad, 2007 [76]	Incarcerated	–	58.5	–	NA	–	–	401 (401,0)	17 (4.0)	17 (4.0)	–	–
30	Davoodian, 2009 [77]	Incarcerated	Mean (SD): 35.4 (8.4)	–	–	NA	–	–	252 (252,0)	12 (4.8)	12 (4.8)	–	HCV: 7(2.8) HIV: 3(1.2); HCV-HIV: 3(1.2)
31	Tavakkoli, 2008 [78]	Mixed	30–44 years: 61.4%	62.0	–	74.5	Homo/bi-sexual: 27.3	Tattoo: 52.5	518 (464, 54)	19 (3.7)	17 (3.7)	2 (3.7)	HBcAb: 317 (61.1); HbeAg: 12 (2.3)
32	Rowhani-Rahbar, 2004 [79]	Incarcerated	Mean: 32.8	48.5	–	NA	Multiple partners: 40.6	Tattoo: 57.4	101 (101,0)	3 (2.9)	3 (2.9)	–	–
33	Masaud, 1996 [80]	Incarcerated	Mean: 34.0	–	–	NA	–	–	88 (48, 40)	5 (5.7)	5 (10.4)	0 (0)	HBeAg: 0 (0); HBeAb: 25 (28.5)

*UK* unknown, *NA* not applicable, *MSM* men having sex with men, *L6M* last 6 months, *DTC* drug treatment centre, *DIC* drop-in centre

^a^HBV positive

^b^HBsAg and HBcAb positive cases

The estimated prevalence of HBV infection in the subgroups of PWID is presented in Table 4. In a descending order, the pooled prevalence of HBsAg positive cases in different recruitment settings was estimated as following: 7.1% (95% CI 5.1–9.7) in treatment centres; 4.7% (95% CI 3.4–6.4) in prisons; 4.5% (95% CI 1.8–10.2) in community settings; 4.3% (95% CI 1.0–14.6) in mixed settings, and 3.3% (95% CI 2.3–4.6) in drop-in centres.

**Table 3:** Findings of studies on HBV prevalence among non-injecting people who use drugs in Iran^a^

No	Author, year	Recruitment setting	Age characteristics	History of incarceration (%)	Sample size (Male, Female)	HBsAg positive cases (%)		
						Total	Male	Female
1	Moradi, 2019 [49]	Incarcerated	–	NA	3917 (UK)	120 (3.0)	–	–
2	Moradi, 2018 [50]	Incarcerated	–	NA	3400 (UK)	95 (2.8)	–	–
3	Ziaee, 2016 [51]	Community	–	–	139 (UK)	5 (3.6)	–	–
4	Mohammadkhani-Ghiasvand, 2016 [56]	DTC	–	–	186 (UK)	3 (1.3)	–	–
5	Teimori, 2011 [59]	DTC	Mean: 35.2 Range: 20–54	1.3	66 (0, 66)	0 (0)	–	0 (0)
6	Nokhodian, 2012 [63]	Incarcerated	–	–	44 (0, 44)	0 (0)	–	0 (0)
7	Khajedaluee, 2016 [65]	Incarcerated	–	–	495 (UK)	16 (3.2)	–	–
8	Mardani, 2009 [70]	Incarcerated	–	–	164 (UK)	6 (3.7)	–	–
9	Talaei, 2007 [71]	Hospital	–	–	126 (UK)	2 (1.6)	–	–
10	Azarkar, 2007 [73]	Incarcerated	–	–	140 (UK)	6 (4.3)	–	–
11	Khodadadizadeh, 2006 [75]	DTC	–	–	149 (141, 8)	2 (3.7)	1 (0.7)	1 (12.5)

*NA* not applicable, *UK* unknown, *DTC *drug treatment center

^a^No study reported data on the history of high-risk sexual relationships, tattooing, cupping and co-infection with HCV or HIV

Four studies reported the co-infection of HBV, HCV, and HIV among PWID, resulting in a pooled prevalence of 2.9% (95% CI 1.2–6.5; *N* = 1362). The same studies also reported HBV and HCV co-infection, which resulted in a pooled prevalence of 7.0% (95% CI 3.1–15.3). Three studies with a sample size of 1266 PWID assessed HBV and HIV co-infection, and the pooled estimate was 4.7% (95% CI 1.8–11.6).

**Fig. 3:**
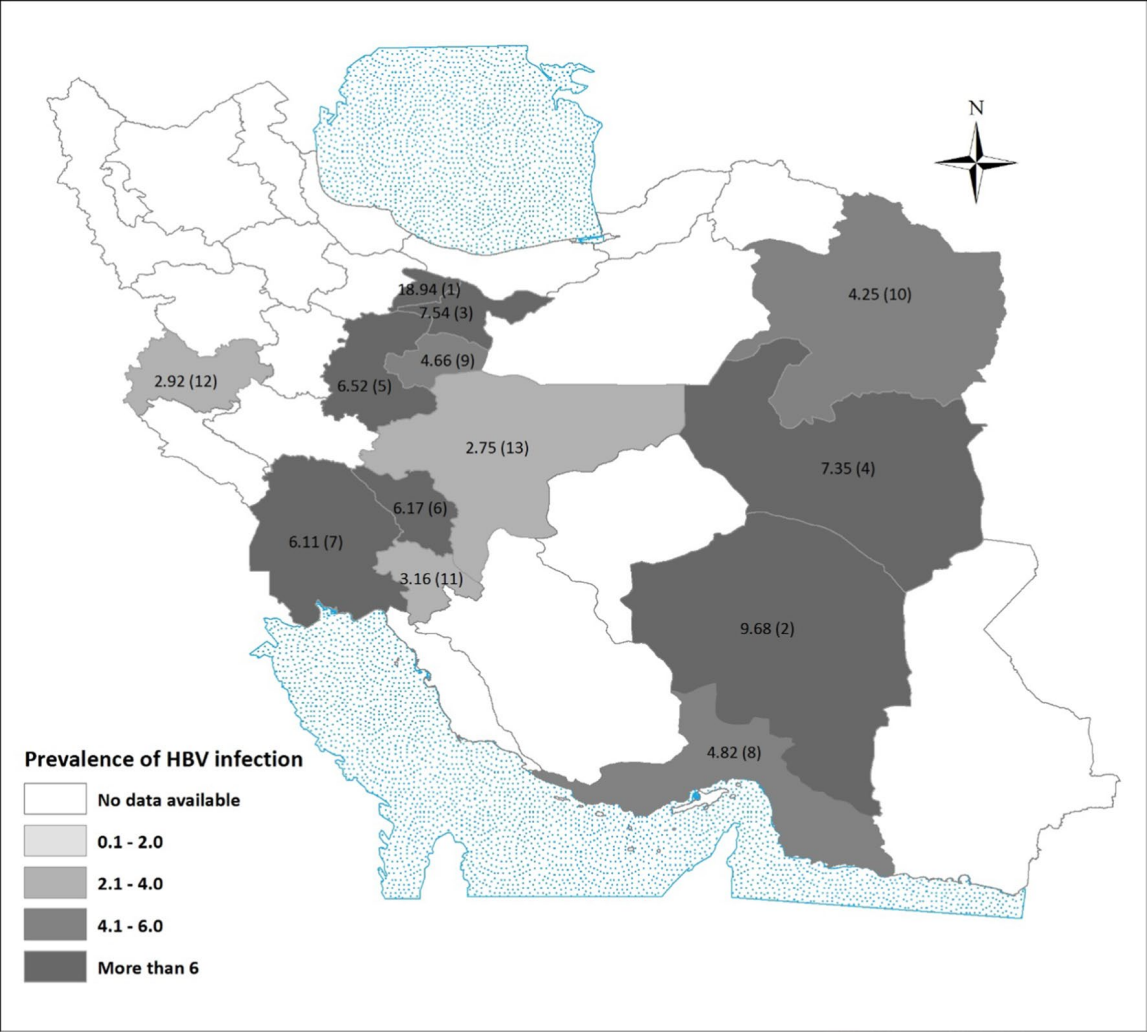
The pooled prevalence of HBV among PWID in different provinces. 1: Alborz, One study, *N* = 132; 2: Kerman, One study, *N* = 31; 3: Tehran, 6 studies, *N* = 2085; 4: South Khorasan, 2 studies, *N* = 68; 5: Markazi, 2 studies, *N* = 253; 6: Charmahal and Bakhtiari, 2 studies, *N* = 162; 7: Khuzestan, One study, *N* = 131; 8: Hormozgan, One study, *N* = 249; 9: Ghom, One study, *N* = 644; 10: Khorasan Razavi, 2 studies, *N* = 212; 11: Kohkiloyeh & Boyerahmad, One study, *N* = 158; 12: Kermanshah, 2 studies, *N* = 616; 13: Isfahan, 6 studies, *N* = 3020

Among different provinces, the highest prevalence of HBV in PWID was 18.9% (95% CI 13.1–26.5, *N* = 132, one study) and was reported from Alborz. Isfahan with a pooled prevalence of 2.7% (95% CI 1.5–4.9, *N* = 3020, 6 studies) had the lowest prevalence. There were no separate data regarding HBV prevalence among PWID in the other 18 provinces (Fig. 3).

The pooled odds ratio of HBV infection among PWID versus non-injecting PWUD is estimated at 1.70 (95% CI 0.90–3.21, *p* value = 0.10). The estimated pooled odds ratios for the association of HBV infection in the PWID and potential risk factors are shown in Table 5. The odds of hepatitis B in PWID with a history of imprisonment is significantly higher than those without a history of imprisonment (OR 1.72, 95% CI 1.29–2.30, *p* value = 0.000). However, in terms of odds ratio, there were no significant association between HBV and the definition of injecting drug use, recruitment setting, gender, age, marital status, employment status, residence, lifetime history of needle sharing, lifetime history of needle sharing in prison, history of men having sex with men, history of extramarital relationships and history of tattooing in PWID participants.

**Table 4:** HBV pooled prevalence across subgroups of PWID

Subgroup	PWID (N)	Studies (N)	Pooled HBV prevalence % (%95 CI)	*I*^2^ (%)
*Recruitment setting*				
Treatment centre	479	7	7.1 (5.1–9.7)	0
Prisons	4740	14	4.7 (3.4–6.4)	79.0
Community	1743	4	4.5 (1.8–10.2)	37.4
Mixed settings	1840	4	4.3 (1.0–14.6)	96.6
Drop-in centres	1003	4	3.3 (2.3–4.6)	0
*Definition of PWID*				
Lifetime	4193	8	3.4 (2.7–4.1)	16.6
Current	2670	10	4.9 (2.8–8.3)	89.2
Unknown	2942	15	5.7 (3.9–8.3)	78.6
*Gender*				
Male	5621	20	5.1 (3.3–7.7)	92.9
Female	199	9	2.9 (0.04–17.2)	77.5
*Age*				
Older	864	3	5.5 (4.1–7.4)	9.6
Youth^a^	206	3	1.4 (0.04–4.4)	0
*Marital status*				
Married^b^	950	5	4.1 (0.08–18.0)	96.2
Never married	1422	5	3.9 (0.06–19.5)	97.0
*Employment status*				
Employed	650	3	9.6 (1.3–44.4)	96.3
Unemployed	619	3	6.7 (0.05–49.7)	89.9
*Current residence*				
Not homeless	969	2	14.6 (1.6–63.7)	98.6
Homeless	492	2	12.7 (1.2–63.0)	95.7
*Lifetime history of imprisonment*				
No	610	6	1.8 (0.02–12.3)	91.1
Yes^c^	6269	20	5.2 (3.3–7.9)	92.3
*Lifetime sharing needle and syringe*				
No	1718	7	2.9 (0.08–9.3)	95.1
Yes	1540	7	7.0 (2.4–18.3)	95.1
*Lifetime sharing needle and syringe in prison*				
No	810	2	9.8 (0.5–70.2)	89.6
Yes	154	2	37.2 (17.1–63.1)	75.6
*Ever MSM*				
No	601	3	2.1 (0.07–5.9)	67.5
Yes	173	3	1.1 (0.01–7.3)	42.5
*Extramarital relationship*				
No	848	2	5.5 (0.01–76.0)	93.8
Yes^d^	316	2	10.8 (0.06–68.7)	88.2
*Ever tattoo*				
No	502	4	1.7 (0.09–3.4)	0
Yes	624	4	2.9 (1.1–7.5)	66.2
*Coinfection*				
HCV, HIV	1362	4	2.9 (1.2–6.5)	73.5
HCV	1362	4	7.0 (3.1–15.3)	89.5
HIV	1266	3	4.7 (1.8–11.6)	88.5

^a^Two studies defined youth under 25 years old and the other one under 30 years old

^b^Three studies only married, two studies married, divorced or widowed

^c^Including those studies recruiting samples from prisons

^d^One study defined as an extramarital relationship without protection in the last 6 months; the other defined as an ever extramarital relationship

**Table 5:** Pooled odds ratios for HBV infection in PWID

Subgroup	Studies (N)	Pooled OR (%95 CI)	*p* value	*I*^2^ (%)
Recruitment setting	33			79.5
Community		1		
Drop-in setting		1.06 (0.33–3.44)	0.91	
Treatment centre		2.24 (0.75–6.66)	0.14	
Prison		1.49 (0.56–3.96)	0.41	
Mixed settings		1.51 (0.48–4.68)	0.47	
Definition of PWID	33			82.3
Current		1		
Lifetime		0.61 (0.30–1.25)	0.17	
Unknown		1.12 (0.61–2.06)	0.71	
Gender (male vs. female)	7	1.48 (0.80–2.73)	0.21	0
Age (youth vs. older)	3	0.46 (0.14–1.54)	0.21	0
Marital status (married vs. unmarried)	5	1.02 (0.78–1.34)	0.88	0
Employment status (employed vs. unemployed)	3	0.86 (0.49–1.50)	0.59	8.8
Residence (not homeless vs. homeless)	2	0.89 (0.68–1.16)	0.39	0
Lifetime history of imprisonment	6	1.72 (1.29–2.30)	*0.000*	0
Lifetime sharing needle and syringe	7	1.57 (0.82–3.01)	0.17	57.6
Hx of sharing needle and syringe in prison	2	3.98 (0.32–49.24)	0.28	80.9
Ever MSM	3	1.52 (0.12–19.94)	0.75	46.6
Extramarital relationship	2	0.96 (0.54–1.73)	0.90	6.6
Ever tattoo	4	1.58 (0.64–3.92)	0.32	0

*MSM* Men having sex with men

### HBV prevalence among non-injecting PWUD

**Fig. 4:**
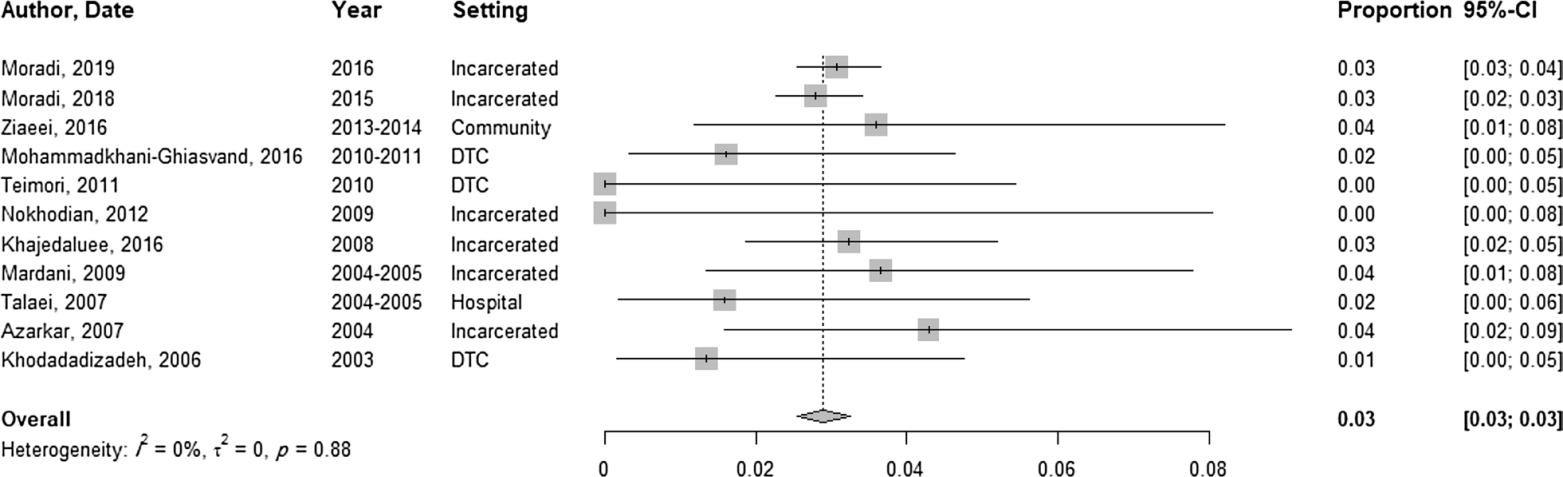
The pooled prevalence of HBV among non-injecting PWUD

The total sample for non-injecting PWUD tested for HBsAg was 8826 (11 studies), and the prevalence of positive HBsAg was between 0 and 4.3% (Table 5). The pooled prevalence in non-injecting PWUD was 2.9% (95% CI 2.5–3.2, *I*^2^ = 0%) (Fig. 4). In non-injecting PWUD, only three studies reported prevalence by gender. Only one study reported a 0.7% (*N* = 149) prevalence of HBsAg among male non-injecting PWUD. The pooled prevalence of HBV among female non-injecting PWUD was estimated to be 0.06% (95% CI 0.0–13.7, *I*^2^ = 42.1%, 3 studies, *N* = 118). The odds of HBV infection were not significantly associated with the recruitment setting.

### Time trend analysis

**Fig. 5:**
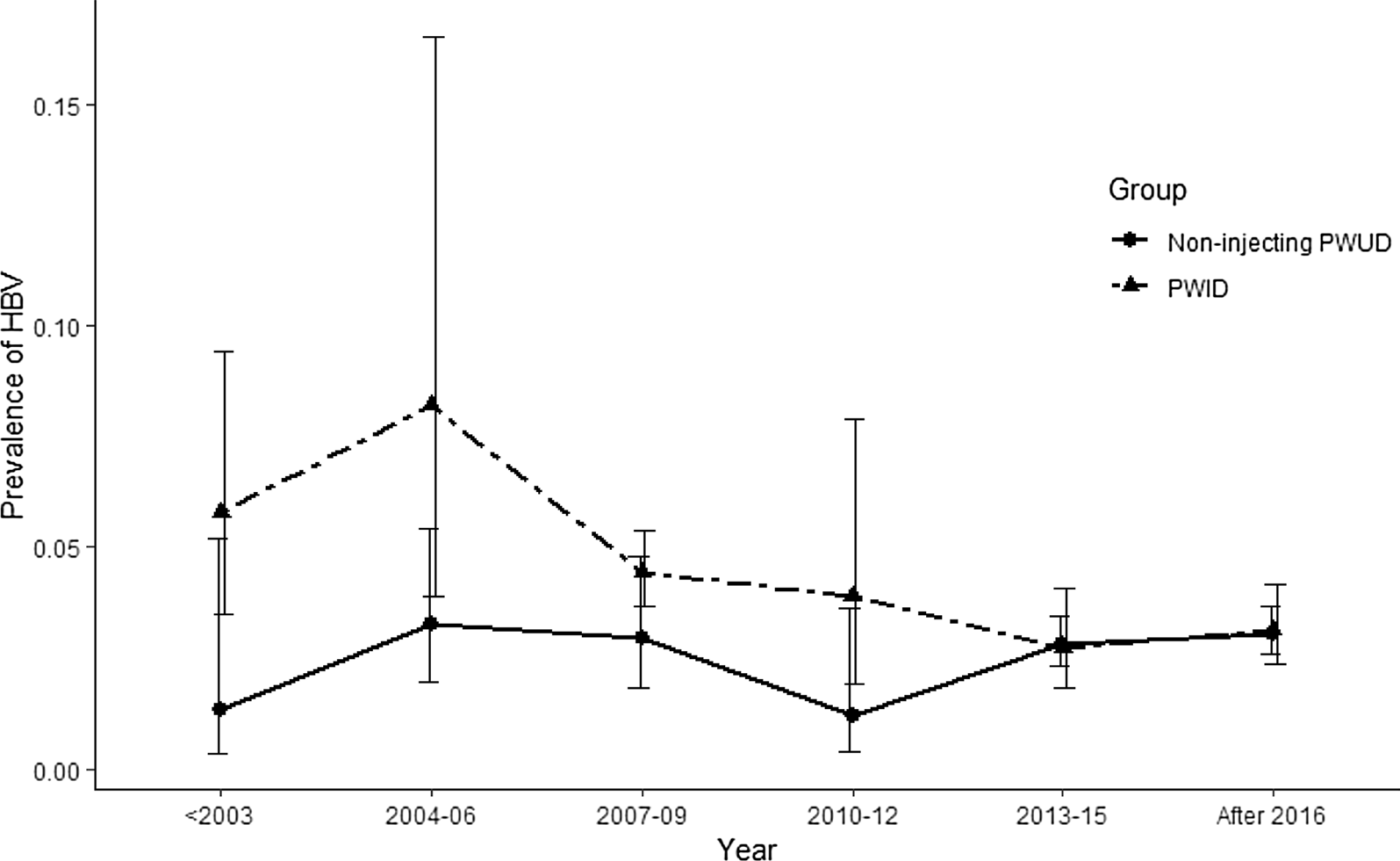
Trend of HBV prevalence among PWID and non-injecting PWUD

The trends of HBV prevalence among PWID and non-injecting PWUD over time are presented in Fig. 5 and Table 6. HBV pooled prevalence among PWID seems to has increased from 5.8% (95% CI 3.5–9.4) in 2003 and before, to 8.2% (95% CI 3.9–16.5) in 2004–2006; however, this increase was not significant (*b* = −0.06; *p* value = 0.30). Afterwards, the pooled HBV prevalence has dropped significantly to 3.1% (95% CI 2.3–4.1) in 2016 and later (*b* = −0.07; *p* value = 0.05). The pooled prevalence of HBV among non-injecting PWUD seems to have increased from 2003 and before until 2004–2006 and then decreased until 2012 and increased afterwards; nonetheless, none of these trends among non-injecting PWUD was statistically significant (*p* value = 0.23, 0.17, 0.12, respectively).

**Table 6:** HBV prevalence and heterogeneity in different periods

Year	No. of studies	Sample size (Total)	HBV prevalence % (95 CI)	*I*^2^ (%)
*PWID*				
≤ 2003	7	84	5.8 (3.5–9.4)	2
2004–2006	4	2093	8.2 (3.9–16.5)	2
2007–2009	8	2231	4.4 (3.7–5.4)	0
2010–2012	7	1682	3.9 (1.9–7.9)	9
2013–2015	4	845	2.7 (1.8–4.1)	0
≥ 2016	3	1434	3.1 (2.3–4.1)	0
*Non-injecting PWUD*				
≤ 2003	1	149	1.3 (0.3–5.2)	−
2004–2006	3	430	3.3 (1.9–5.4)	0
2007–2009	2	539	3.0 (1.8–4.8)	0
2010–2012	2	252	1.2 (0.4–3.6)	0
2013–2015	2	3539	2.8 (2.3–3.4)	0
≥ 2016	1	3917	3.0 (2.6–3.6)	−

Model: Random Effects Model

### Heterogeneity of the studies

For finding the source of heterogeneity between the studies on PWID, meta-regression analyses were performed and none of the following potential predictors had significant influence on HBV prevalence: number of unfulfilled quality criteria (*p* value = 0.3), the definition of injection drug use as lifetime or unknown versus current (*p* value = 0.2), and setting for recruiting the sample (all versus community; *p* value = 0.5). The results of meta-regression on studies on non-injecting PWUD assessing potential predictors were also not significant: the number of unfulfilled quality criteria (*p* value = 0.9), the definition of non-injection as lifetime or unknown versus current (*p* value = 0.9), and setting for recruiting the sample (all versus community; *p* value = 0.2).

## Discussion

We found a pooled prevalence of 4.8% for HBV among PWID in Iran; 5.1% in male and 2.9% in female subgroups. One previous systematic review on Iranian high-risk population groups, including other vulnerable groups such as sex workers and prisoners, also estimated a prevalence of 4.8% HBV infection [22]. In a systematic review published by Degenhardt et al. in 2017, the prevalence of HBV among PWID was investigated. Globally, it has been estimated that 9% of PWID are positive for HBsAg and the estimate for the Middle East and North African region is 8.1% [4]. This paper reviewed studies published from 2011 to June 2017 and included six studies from Iran, three of which recruited the participants from infectious wards or HIV care clinics. The review reported a pooled HBV prevalence of 3.9% (95% CI 2.9–4.9) among Iranian PWID.

In our study, the odds of HBV were significantly higher among PWID with a history of imprisonment. Our results are in concordance with the previous literature [3, 23–26]. In intermediate-prevalence areas for HBV, including Iran, the predominant mode of HBV transmission is through sexual contact and injecting drug use [27, 28]. Our study showed a higher prevalence of HBV among PWID with a history of sharing needles and syringes and high-risk sexual behaviours (such as extramarital relationship or MSM). However, we found no significant association between these factors and HBV infection in terms of odds ratio, which may be due to small sample sizes and power.

The pooled prevalence of HBV among Iranian non-injecting PWUD was 2.9%. Due to lack of data, the associated risk factors could not be further investigated. Two recent systematic reviews estimated the prevalence of HBV infection among the Iranian general population to be 2.2 and 3.0% [29, 30]; seemingly not lower than the prevalence in non-injecting PWUD in our study. Presumably, high-risk sexual behaviours among non-injecting PWUD are similar to the general population in Iran.

We performed a trend analysis of HBV prevalence based on the implementation year. Although the prevalence of HBV among PWID increased slightly before 2006, this increase was not found significant. Since then, HBV prevalence among PWID has decreased significantly and reached 3.1% in recent years, which is not considerably higher than the general population. Similarly, the HBV prevalence declined since 2006 in the Iranian general population [29]. However, none of the trends seen for HBV prevalence among non-injecting PWUD was statistically significant.

The recent significant reduction in the prevalence of HBV among PWID may be attributed to public health measures implemented against HIV and hepatitis. Harm reduction measures were initiated in 2002 and scaled up in 2005 and included needle and syringe programs and opioid substitution treatment [31–34]. Needle and syringe and opioid substitution treatment programs continued to grow nationally in the following decade [35, 36]; hypothetically, being the cause for the current reduction in HBV prevalence among PWID. A similar trend has been reported for HIV infection among PWID [15]. It seems that these changes are the results of the decrease in high-risk behaviours. In a national study in 2010, 36.9% reported a positive history of unsafe injection in the previous month [37]. In the latest national survey on drug users in 2018, only 22% of the PWID reported lending or/borrowing used syringes in the previous year [12]. HBV vaccination coverage was initiated in 1992 targeting newborns; however, there has been no vaccination program specifically for high-risk groups including PWUD. Another review from Iran has shown that the weighted mean age of first injection is 25.8 [38]. As a result, this policy measure may have partly targeted the current generation of PWID and a higher impact would be expected in the future.

For maintaining the reduction in HBV prevalence in the PWID, it seems that sustaining needle syringe and opioid substitution treatment programs are vital. History of imprisonment was found as a correlate of HBV infection among PWID, which necessitates a further expansion of harm reduction measures such as needle and syringe programs and HBV diagnostic and treatment programs in the prisons’ health system [39, 40]. Targeted HBV vaccination for PWID, whether with the regular or accelerated protocol is a recommended intervention for HBV control [41, 42]. Viral hepatitis, including HBV, results in higher mortality than HIV [43]; however, globally it has been neglected in the majority of the policies [3]. The WHO's package for ending viral hepatitis includes a high coverage of needle syringe program, opioid substitution treatments and treatment for other drug types, diagnosis and treatment of viral hepatitis for PWUD [1]. In spite of expansion in harm reduction services in Iran, the coverage is still low to moderate [44].

Though the pooled prevalence was higher among the male subgroup of PWID compared with female subgroup, the difference was not statistically significant. Most of the studies on non-injecting PWUD did not report gender-specific data; thus, it could not be further interpreted. Furthermore, female-specific risk factors for HBV infection were not investigated in the included studies. Globally, HBV prevalence among female and male PWID subgroups was not also significantly different [45].

We faced notable shortcomings in a number of included studies. There were 9 (25.7%) low-quality studies among total inclusions. Almost 45% of the studies did not report the definition of injecting drug use, and in the others, a variety of definitions were used. Although sexual transmission is the major route of HBV acquisition, only a few studies evaluated the history of high-risk behaviours using various definitions.

There were fewer studies targeting non-injecting PWUD. Further data regarding possible risk factors, such as high-risk sexual contacts, among non-injecting PWUD, were not collected. HBV prevalence among PWUD has not been investigated in several provinces. For instance, there is no study on PWUD in Golestan province, in which there is evidence of a high HBV prevalence among the general population [30]. Rural areas were also completely neglected. Moreover, the association between HBV infection and type of substances has not been investigated. With an increase in methamphetamine use in the country [46], the HBV epidemics among PWUD might be affected. Therefore, there is a need for additional high-quality researches for providing a more accurate picture of HBV prevalence among PWUD subgroups.

We were able to provide pooled HBV prevalence among both injecting and non-injecting PWUD and several subgroups in Iran. However, we faced considerable heterogeneity among PWID. Thus, through subgroup analyses and meta-regression model, we tried to investigate the source of heterogeneity. Yet, the heterogeneity was still considerable among most of the subgroups and we did not identify any source in the meta-regression model. Therefore, the results should be interpreted with caution.

## Conclusion

The prevalence of HBV infection among non-injecting PWUD and even PWID was not considerably higher than the Iranian general population. Although PWID, as well as non-injecting PWUD, are considered as the main high-risk groups for HIV and HCV infections in Iran, it is not the case for HBV infection. A significant decreasing trend was detected for HBV infection among PWID subgroup in recent years. Still, it seems that there are subgroups of PWID, which have not adequately benefited from harm reduction interventions as well as specific preventive measures for HBV. Future programs should more specifically target these high-risk groups.

## Data Availability

All data generated or analysed during this study are included in this published article.

## Abbreviations

CI: Confidence interval
DIC: Drop-in centre
DTC: Drug treatment centre
HBsAg: Hepatitis B Virus surface antigen
HBV: Hepatitis B Virus
HCV: Hepatitis C virus
HIV: Human immunodeficiency virus
L6M: Last 6 months
MSM: Men having sex with men
NA: Not applicable
PRISMA: Preferred Reporting Items for Systematic Reviews and Meta-analysis
PWID: People who inject drugs
PWUD: People who use drugs
UK: Unknown
WHO: World Health Organization
